# IRF4-Dependent and IRF4-Independent Pathways Contribute to DC Dysfunction in Lupus

**DOI:** 10.1371/journal.pone.0141927

**Published:** 2015-11-06

**Authors:** Michela Manni, Sanjay Gupta, Briana G. Nixon, Casey T. Weaver, Rolf Jessberger, Alessandra B. Pernis

**Affiliations:** 1 Autoimmunity and Inflammation Program, Hospital for Special Surgery, New York, New York, United States of America; 2 Graduate Program in Immunology and Microbial Pathogenesis, Weill Cornell Graduate School of Medical Sciences, New York, New York, United States of America; 3 Department of Pathology, University of Alabama at Birmingham, Birmingham, Alabama, United States of America; 4 Institute of Physiological Chemistry, Technische Universität Dresden, Dresden, Germany; 5 Department of Medicine, Weill Cornell Medical College, Cornell University, New York, New York, United States of America; INSERM-Université Paris-Sud, FRANCE

## Abstract

Interferon Regulatory Factors (IRFs) play fundamental roles in dendritic cell (DC) differentiation and function. In particular, IRFs are critical transducers of TLR signaling and dysregulation in this family of factors is associated with the development of autoimmune disorders such as Systemic Lupus Erythematosus (SLE). While several IRFs are expressed in DCs their relative contribution to the aberrant phenotypic and functional characteristics that DCs acquire in autoimmune disease has not been fully delineated. Mice deficient in both DEF6 and SWAP-70 (= Double-knock-out or DKO mice), two members of a unique family of molecules that restrain IRF4 function, spontaneously develop a lupus-like disease. Although autoimmunity in DKO mice is accompanied by dysregulated IRF4 activity in both T and B cells, SWAP-70 is also known to regulate multiple aspects of DC biology leading us to directly evaluate DC development and function in these mice. By monitoring Blimp1 expression and IL-10 competency in DKO mice we demonstrate that DCs in these mice exhibit dysregulated IL-10 production, which is accompanied by aberrant Blimp1 expression in the spleen but not in the peripheral lymph nodes. We furthermore show that DCs from these mice are hyper-responsive to multiple TLR ligands and that IRF4 plays a differential role in in these responses by being required for the TLR4-mediated but not the TLR9-mediated upregulation of IL-10 expression. Thus, DC dysfunction in lupus-prone mice relies on both IRF4-dependent and IRF4-independent pathways.

## Introduction

The Interferon Regulatory Factor (IRF) family of transcription factors plays a key role in the control of both innate and adaptive immune responses and aberrancies in the expression and/or function of IRF family members have been associated with the development of several autoimmune diseases including Systemic Lupus Erythematosus (SLE) [1–4]. The IRFs share a conserved N-terminal helix-turn-helix DNA binding domain (DBD) containing five conserved tryptophan residues separated by 10–18 amino acids [5]. The IRF C-terminal region contains a domain termed the IRF-associated domain (IAD), which mediates protein-protein interactions with other IRFs and additional transcriptional regulators [5]. Given that several IRFs can be expressed within the same cell, the expression of IRF-regulated genes is determined by a complex interplay dictated by the affinity of distinct IRFs for specific DNA elements, their ability to associate with additional partners such as Ets and BATF proteins, as well as antagonistic and/or synergistic interactions amongst different IRFs [6, 7].

Amongst IRF family members, IRF4 has emerged as a key and multifaceted regulator of adaptive immune responses. IRF4 was originally recognized as a major controller of critical B cell processes like class switch recombination (CSR) and plasma cell differentiation via its ability to regulate the expression of AID (Activation-induced DNA-cytosine deaminase) and Blimp1, respectively [8, 9]. IRF4 also controls the differentiation of T_H_2, T_H_9, T_FH_ and T_H_17 cells and is absolutely required for the production of IL-17 and IL-21, which mediate pathogenic effects in several autoimmune diseases [10–16]. The presence of IRF4 is also necessary for the function of Tregs and their ability to produce IL-10, an effect that relies on the capacity of IRF4 to upregulate the expression of Blimp1 and to cooperate with this transcription factor [17].

The role of IRF4 in immune responses is not solely confined to T and B cells. Dendritic cells (DCs) also depend on IRF4 for their differentiation and function [4]. Indeed IRF4 promotes the differentiation of CD11b^+^ DCs while IRF8, an IRF family member that shares the greatest similarity with IRF4, is necessary for the development of CD11b^-^ DCs and plasmacytoid DCs (pDCs) [18–20]. One of the key roles of IRF4 in CD11b^+^ DCs is to regulate the expression of components of the MHC class II antigen presentation pathway [21]. In addition to controlling the development of CD11b^+^ DCs, IRF4 has been shown to exert a number of selective effects on DC function. IRF4 regulates the migration of DCs in the skin as well as the differentiation of specialized DC subsets, which can promote either T_H_17 or T_H_2 responses [22–26]. The T_H_2-promoting DC subset is characterized by the expression of high levels of PDL2 and the production of IL-10, whose regulatory regions in DCs are co-bound by IRF4 and the Ets protein PU.1 [17, 23].

Given the essential and complex role of IRF4 in innate and adaptive immune responses, it is not surprising that this transcription factor is becoming recognized as a key player in lupus pathogenesis. Lack of IRF4 in B6.lpr mice abrogates the development of lupus nephritis, an effect that occurs despite increases in the production of proinflammatory cytokines [27]. Furthermore, mice deficient in DEF6 and SWAP-70 (= Double-knock-out or DKO mice), two members of a unique family of molecules that restrain IRF4 function, spontaneously develop lupus on a C57BL/6 background. Autoimmunity in DKO mice is accompanied by dysregulated IRF4 activity in both T and B cells [28]. Notably, CD4^+^ T cells in DKO mice aberrantly produce IL-17 and IL-21 due to the deregulated ability of IRF4 to drive the production of these cytokines [28–30]. This effect has been linked to increased phosphorylation of IRF4 by the serine-threonine kinase, ROCK2 [29]. The ROCK-IRF4 axis may play a broad pathogenic role in lupus since administration of a ROCK inhibitor, Fasudil, ameliorated lupus pathogenesis in two additional mouse models of SLE, MRL/lpr mice and NZB/W F1 mice [29, 31]. Enhanced ROCK activity has furthermore been observed in PBMCs from SLE patients [32] and the recent development of both panROCK inhibitors and selective ROCK2 inhibitors [33] has opened the possibility that targeting the ROCK-IRF4 axis may represent a novel therapeutic approach for SLE.

In view of the emerging role of IRF4 in lupus pathogenesis, a better understanding of its precise contribution to the broader cellular abnormalities that accompany the development of lupus could provide important new insights into the pathogenesis of this disease. Given the importance of IRF4 in DC differentiation and activation and given that SWAP-70 is known to regulate multiple aspects of DC biology, including MHCII surface localization and the spontaneous maturation of DCs [34–38], here we explored whether DEF6 and SWAP-70 regulate IRF4 activity in DCs and investigated the impact exerted by IRF4 on DC dysfunction in these mice.

## Materials and Methods

### Mice

DEF6 and Swap-70 double knock out (DKO) mice were generated by crossing Def6^trap/trap^ mice with Swap70ko mice that had been backcrossed onto the C57BL/6 background as previously described [28]. Blimp1-YFP-10BiT double reporter mouse were obtained from S. Kaech and have been described previously [39, 40]. Blimp1-YFP-10BiT mice were crossed with DKO mice to generate Blimp1-YFP-10BiT DKO mice. CD11c-Cre mice [41] were purchased from Jackson Laboratory and crossed with DKO mice to generate CD11c-Cre DKO mice. IRF4^fl/fl^ were obtained from U. Klein [8] and crossed with CD11c-Cre DKO to obtain CD11c-Cre IRF4^fl/fl^ DKO mice. All mice were maintained under specific pathogen-free conditions. The experimental protocols were approved by the Institutional Animal Care and Use Committee at the Hospital for Special Surgery.

### Antibodies and Flow cytometry

Single cell suspensions were prepared from spleen and lymph nodes, treated with red blood cell lysis (R&D) and blocked with Fc-Block prior to staining in cold PBS with 1% FBS with antibodies anti-CD11c (Biolegend), IAb (Biolegend), CD8 (Biolegend), CD11b (Biolegend), CD86 (Biolegend), PDL2 (Biolegend), PDL1 (Biolegend), B220 (Biolegend), PDCA-1 (Biolegend), Thy1.1 (Biolegend), CD44 (Biolegend), CD4 (Biolegend), PD1 (ebiosciences), CXCR5 (BD Biosciences), GL7 (ebiosciences) and CD138 (Biolegend). The Foxp3 staining kit (eBiosciences) was used for intracellular staining of Foxp3 (eBiosciences). Data were acquired by FACSCanto (BD Biosciences) and analyzed using FlowJo Software (Tree Star).

### In vitro bone marrow derived DC generation and stimulation

Bone marrow cells were cultured at a concentration of 2.5 x 10^6^ cells/ml for 7 days in complete RPMI 1640 culture media containing 10% FBS, 50μM **β**-Mercaptoethanol (Sigma), 100U/ml Penicillin, 100**μ**g/ml Streptomycin, 2mM L-Glutamin and 25mM Hepes (all from Corning) in presence of 20ng/ml GM-CSF (Peprotech). Half of the media was replaced at day 3 and 5. At day 7 CD11c^+^ DC were selected by CD11c positivity using magnetic microbeads in a magnetic cell sorting system (Miltenyi). One million cell per ml were plated in complete media in absence of GM-CSF and left untouched or stimulated for 24h with 0.1**μ**g/ml LPS (Sigma), 3**μ**M CpG ODN 2395 (Invivogen) or 3**μ**g/ml Imiquimod (Invivogen).

### Real-Time RT-PCR

Total RNA was isolated from cells using RNeasy Plus Mini kit (Qiagen) and reverse transcribed using the iScript cDNA synthesis kit (Biorad). Real-Time RT PCR was performed using the iTaq Universal SYBR Green Supermix (Biorad). Gene expression was calculated using the **ΔΔ**Ct method and normalized to the housekeeping gene Cyclophilin A.

### ELISA

IL-10 and IFN-**β** concentrations in the culture supernatants were quantified by ELISA Max Standard Set (Biolegend) and Legend Max ELISA kit (Biolegend), respectively.

### Cell Extracts and Western Blotting

Whole cell extracts were prepared using 1% Triton-x 100. Blots were probed with antibodies against DEF6, SWAP70, IRF4 and IRF8. **β**-Actin was used as loading control.

### Statistical Analysis

Statistical significance was determined by unpaired two-tailed Student’s t test or ANOVA followed by *post hoc* Tukey test using Prism software (GraphPad).

## Results

### Relative expansion of CD11b^+^ DCs in DKO mice

To start evaluating whether DEF6 and SWAP-70 might control IRF4 function in CD11b^+^DCs we first assessed the expression of these molecules in GM-CSF-derived bone-marrow DCs (BMDCs), a culture system that leads to the generation of DCs closely resembling CD11b^+^DC [21]. In addition to SWAP-70, whose expression had previously been reported in this compartment [42], BMDCs were also found to express DEF6 (S1 Fig). Interestingly, the expression of DEF6 was increased upon stimulation of BMDCs with ligands for TLR4, TLR7, or TLR9 while expression of SWAP-70 was not significantly altered (S1 Fig).

**Fig 1:**
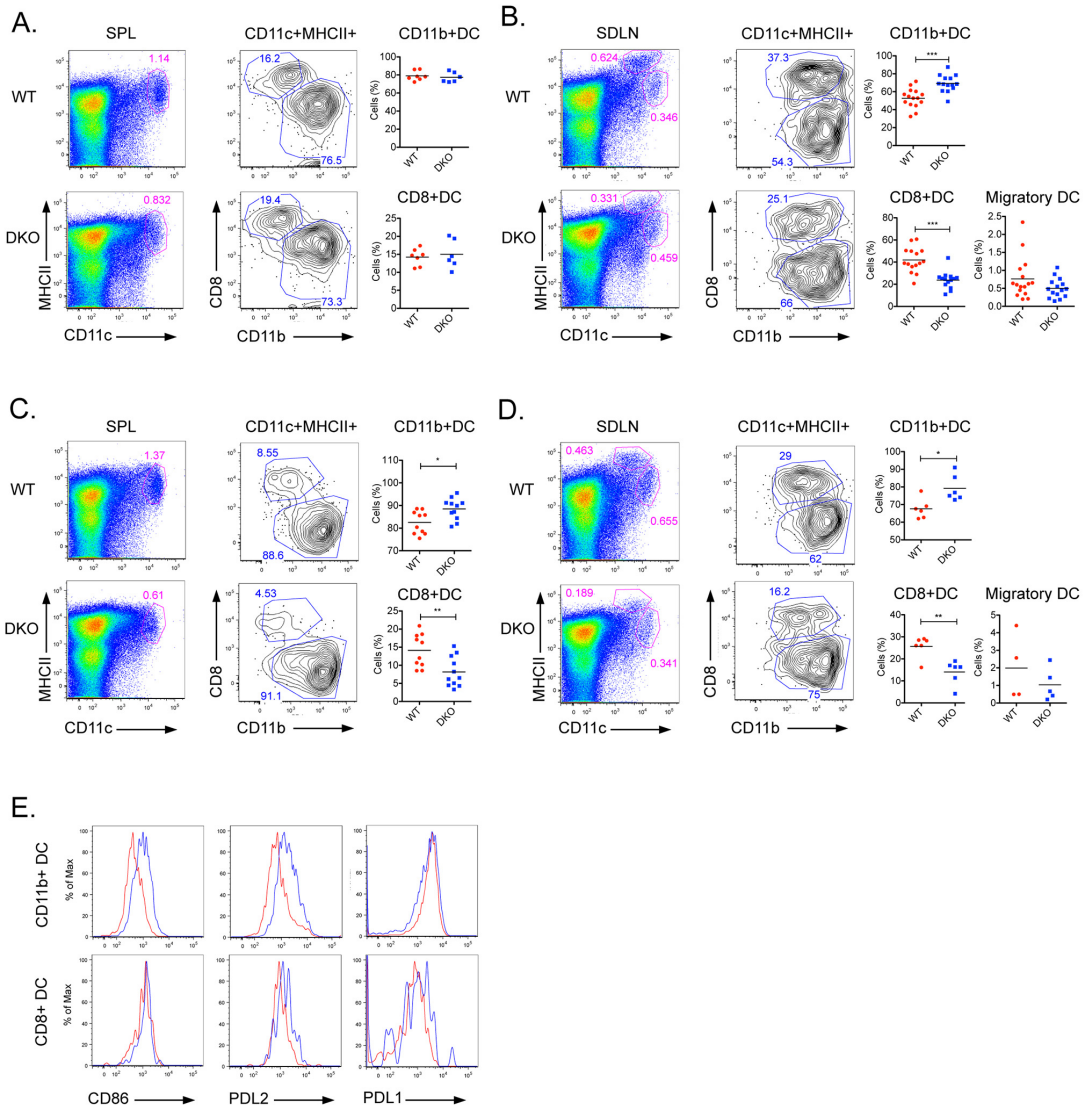
Relative expansion of CD11b^+^ DCs in DKO mice. Spleen (SPL) (**A**) and skin draining lymph nodes (SDLN) (**B**) of 8 weeks old WT and DKO female mice were assayed for DC populations by flow cytometry. Splenocytes were gated on MHCII^+^CD11c^+^ conventional DCs and analyzed for the proportion of CD8^+^DCs and CD11b^+^DCs. In SDLNs, MHCII^Hi^CD11c^+^ migratory DCs were also examined. Spleen (**C**) and skin draining lymph nodes (**D**) of >24 weeks old mice were also assayed for DC populations by flow cytometry. Cells were gated on MHCII^+^CD11c^+^B220^-^ conventional DCs and analyzed for the proportion of CD8+DCs and CD11b^+^DCs. In lymph nodes, MHCII^Hi^CD11c^+^ migratory DC frequencies were also examined. Scatter plots show data of individual mice and mean value of at least 3 independent experiments. *: p<0.05; **: p<0.01, ***: p≤0.0001. **(E)** CD86, PDL2 and PDL1 cell surface expression was analyzed by flow cytometry on conventional CD11b^+^DCs and CD8^+^ DCs in the spleen of >24 weeks old WT (red) and DKO (blue) mice. Histograms show relative expression of the indicated marker. Representative data of 2–4 independent experiments with a total of 5–9 mice per group are shown.

Given that DCs express both DEF6 and SWAP-70 we next examined whether the lack of these molecules would affect DC development. No significant differences in the relative distribution or numbers of splenic CD11b^+^DC, CD8^+^DC, or pDCs were observed in young wild-type (wt) and DKO mice (Fig 1A and S1 Fig). Skin draining lymph nodes (SDLNs) of DKO mice instead demonstrated an expansion of CD11b^+^ DCs with a corresponding decrease in the frequency of the CD8^+^ DC population and no significant abnormalities in the migratory DC compartment (Fig 1B and S1 Fig). Aging DKO female mice demonstrated even more striking changes in the relative distribution of CD11b^+^ DCs and CD8^+^ DCs, which could now be observed in both spleens and SDLNs (Fig 1C and 1D). No effects on pDCs were again observed (S1 Fig). Thus, in the absence of DEF6 and SWAP-70, the relative balance between CD11b^+^ DCs and CD8^+^ DCs is skewed in favor of CD11b^+^ DCs.

IRF4 has been implicated in the differentiation of PDL2^+^ DCs [25] prompting us to directly investigate the expression of PDL2 and its homolog PDL1 on wt and DKO DCs. Since higher levels of CD86 are upregulated on DCs in the absence of SWAP-70 [35] we also assessed its expression. Both CD86 and PDL2 were expressed at higher levels on DKO DCs from older mice as compared to wt DCs while the expression of PDL1 on these cells was not affected by the lack of DEF6 and SWAP-70 (Fig 1E). PDL2 upregulation could be primarily observed on CD11b^+^ DCs while neither CD8^+^ DC nor pDCs from DKO mice expressed higher levels of PDL2 or CD86 as compared to wt mice (Fig 1E and S1 Fig).

### Increased production of IL-10 by CD11b^+^ DKO DCs

**Fig 2:**
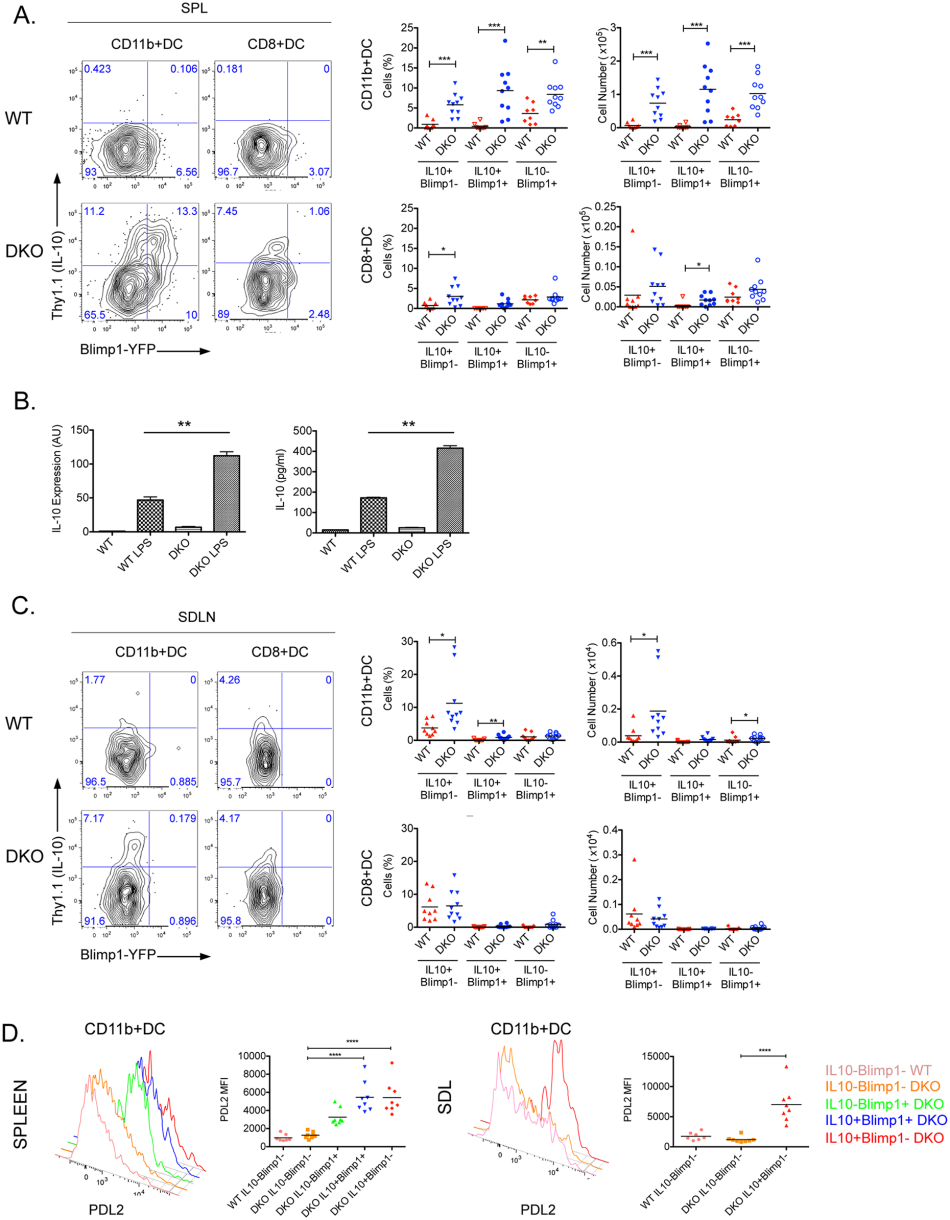
Increased IL-10 production by CD11b^+^ DKO DCs. (**A**) Spleens (SPL) from 14–16 weeks old WT and DKO IL-10 and Blimp1 dual reporter mice were examined by flow cytometry. Splenocytes were gated on MHCII^+^CD11c^+^B220^-^ conventional DCs followed by analysis of YFP (Blimp1) and Thy1.1 (IL-10) expression on CD11b^+^DCs and CD8^+^DCs. Percentages and numbers of Thy1.1^+^YFP^-^, Thy1.1^+^YFP^+^ and Thy1.1^-^YFP^+^ cells are shown. Scatter plots show data of individual mice and mean value of 4 independent experiments. *: p<0.05; **: p<0.01, ***: p<0.001. (**B**) IL-10 production by splenic CD11c^+^ cells stimulated *in vitro* with 0.1 **μ**g/ml LPS for 24 hours was analyzed by qPCR and ELISA. Representative data of 3 independent experiments are shown **: p<0.01. (**C**) Skin draining lymph nodes (SDLN) from 14–16 weeks old WT and DKO IL-10 and Blimp1 dual reporter mice were examined by flow cytometry. Cells were gated on MHCII^+^CD11c^+^B220^-^ conventional DCs followed by analysis of YFP (Blimp1) and Thy1.1 (IL-10) expression on CD11b^+^DCs and CD8^+^DCs. Percentages and numbers of Thy1.1^+^YFP^-^, Thy1.1^+^YFP^+^, and Thy1.1^-^YFP^+^ cells are shown. Scatter plots show data of individual mice and mean value of 4 independent experiments. *: p<0.05; **: p<0.01, ***: p<0.001. (**D**) PDL2 cell surface expression on DCs from WT and DKO IL-10 and Blimp1 dual reporter mice was analyzed by flow cytometry. Splenic and skin draining lymph node (SDLN) cells were gated on MHCII^+^CD11c^+^CD11b^+^ DCs and PDL2 expression was analyzed on Thy1.1^-^YFP^-^, Thy1.1^+^YFP^-^, Thy1.1^+^YFP^+^ and Thy1.1^-^YFP^+^ subsets. Histograms show representative expression of PDL2. Scatter plots show mean florescence intensity (MFI) data of individual mice and mean value of 3 independent experiments: ****: p<0.0001.

The expansion of the DKO CD11b^+^ DC population together with their higher levels of PDL2 supported the idea that the absence of DEF6 and SWAP-70 leads to enhanced IRF4 activity in this compartment. Given that IL-10 is a well-known target of IRF4 in DCs and that, in Tregs, IRF4 cooperates with Blimp1 in driving IL-10 expression, we investigated the Blimp1-IL-10 module in DKO DCs by employing a dual reporter mouse where the expression of Blimp1 and IL-10 competency can be simultaneously tracked by assessing the expression of YFP (for Blimp1) and Thy1.1 (for IL-10) [39, 40]. Neither CD11b^+^ nor CD8^+^ DCs expressed IL-10 in spleens of wt mice (Fig 2A). In contrast, splenic CD11b^+^ DC cells from DKO mice expressed high levels of Thy1.1 (Fig 2A). Enhanced Blimp1 expression accompanied IL-10 competency in a substantial proportion of the CD11b^+^ DKO DCs. To confirm that the higher levels of Thy1.1 detected in splenic DKO DCs indeed correlated with increased IL-10 production, we isolated splenic DCs and restimulated them *in vitro* with LPS (Fig 2B). Consistent with the findings obtained with the reporter mice, DKO DCs produced higher levels of IL-10 than wt DCs. In contrast to CD11b^+^ DCs, only a small percentage of CD8^+^ DKO DCs expressed Thy1.1 (Fig 2A). The levels of Thy1.1 on CD8^+^DKO DCs were furthermore lower than those observed in the CD11b^+^ DKO DCs and were not accompanied by increased Blimp1 expression. Thus splenic CD11b^+^ DKO DCs display an enhanced ability to express both IL-10 and Blimp1.

An analysis of SDLNs again revealed increased IL-10 expression by CD11b^+^ DKO DCs (Fig 2C). However, in contrast to the findings in the spleen, SDLN CD11b^+^ DKO DCs did not express Blimp1. In addition, no upregulation of IL-10 production could be observed in CD8^+^ DKO DCs. Enhanced PDL2 expression could be detected in both splenic and SDLN DCs that upregulated IL-10 expression regardless of their Blimp1 status (Fig 2D). Thus, local factors may regulate the ability of CD11b^+^ DKO DCs to coexpress high levels of IL-10 and Blimp1 versus IL-10 alone.

Given that macrophages have been reported to be able to produce IL-10 in the lupus-prone MRL/lpr mouse [43] we also assessed IL-10 production and Blimp1 expression in this compartment. Splenic macrophages from DKO mice exhibited higher levels of Thy1.1 expression than macrophages from wt mice (S2 Fig). The levels of IL-10 expressed by DKO macrophages were, however, lower than those observed in the CD11b^+^ DKO DCs and DKO macrophages lacked Blimp1. Regional differences were again detected as evidenced by the finding that the enhanced expression of IL-10 by DKO as compared to wt macrophages was primarily detected in the spleen but not in SDLN (S2 Fig).

### Increased TLR-mediated induction of IL-10 and IFNβ expression by DKO BMDCs

**Fig 3:**
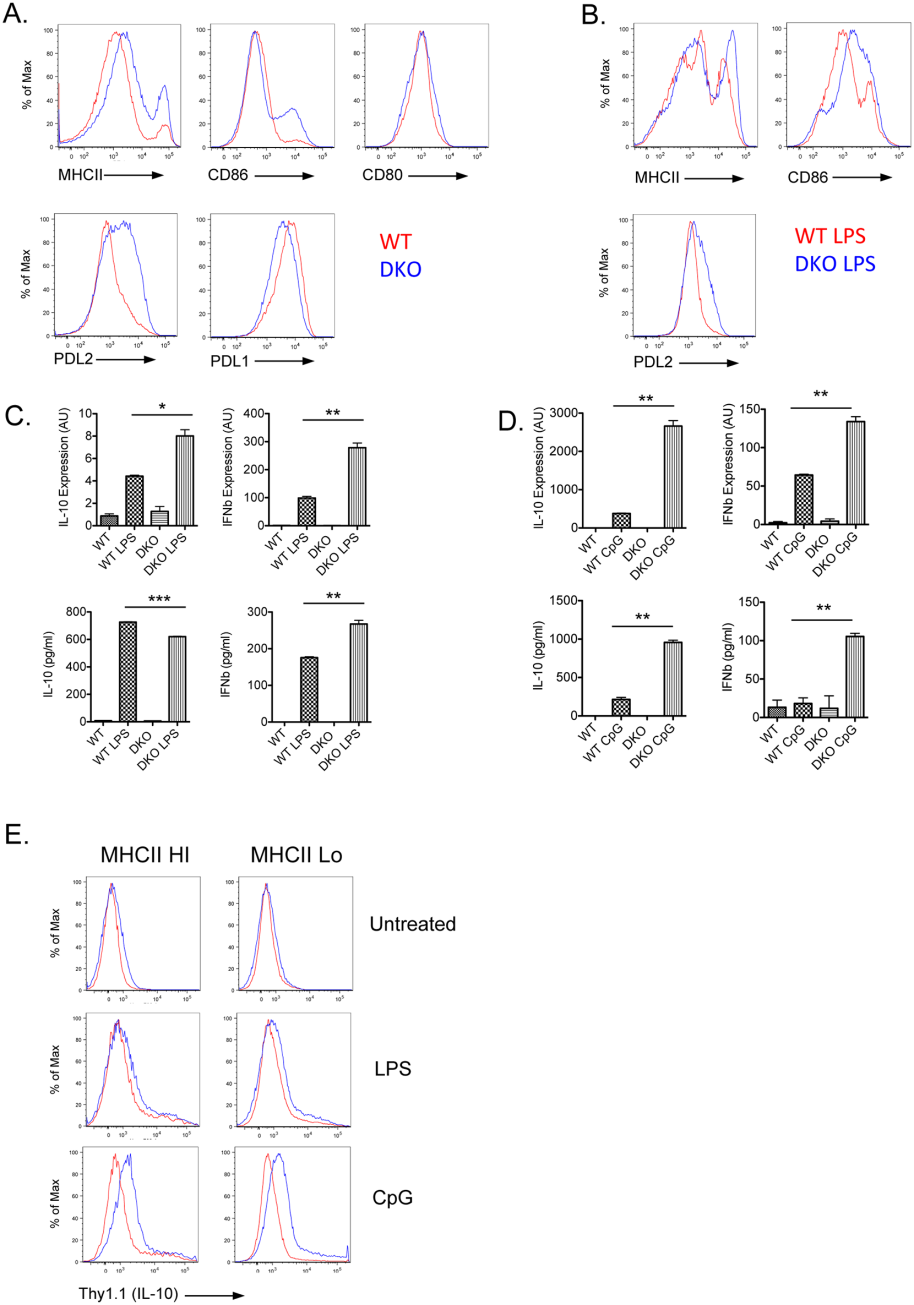
Increased IL-10 and IFNβ expression by TLR-stimulated DKO BMDCs. (**A**) WT and DKO BMDCs were generated *in vitro* in presence of GM-CSF for 7–9 days prior to FACS analysis of MHCII, CD86, CD80, PDL2 and PDL1. Histograms show relative expression of the indicated marker on WT (red) or DKO (blue) CD11c^+^DCs. One representative experiment out of 1 (CD80) or at least 2 independent experiments (MHCII, CD86, PDL2 and PDL1) is shown. **(B-E)** BMDCs were generated *in vitro* for 7 days. CD11c^+^DCs were purified by magnetic sorting followed by *in vitro* stimulation with 0.1**μ**g/ml LPS or 3**μ**M CpG for 24h. (**B**) Cells were harvested and MHCII, CD86 and PDL2 expression on WT and DKO LPS treated CD11c^+^ DCs analyzed by FACS. One representative experiment out of 2 independent experiments is shown. IL-10 and IFN**β** production and gene expression in LPS treated (**C**) or CpG treated (**D**) WT and DKO BMDCs were assayed by ELISA and qPCR. One representative experiment out of at least 3 independent experiments is shown. *: p<0.05; **: p<0.01, ***: p<0.001 (**E**) Thy1.1 expression in BMDCs from IL-10 and Blimp1 dual reporter WT or DKO mice was analyzed by FACS. Histograms show relative expression of Thy1.1 on WT (red) or DKO (blue) CD11c+MHCII^hi^ or CD11c+MHCII^lo^ DCs. One representative experiment out of at least 2 independent experiments is shown.

The enhanced ability of splenic DKO DCs to produce IL-10 in response to LPS suggested that the absence of DEF6 and SWAP-70 might render DCs hyper-responsive to TLR stimulation. To further explore this notion we generated BMDCs from wt and DKO mice. As compared to wt BMDCs and consistent with our previous data on SWAP-70 knock-out mice [35], after 7 days of culture DKO BMDCs expressed higher level of Class II MHC and CD86 but equivalent levels of CD80 (Fig 3A). Consistent with our *in vivo* findings, DKO BMDCs exhibited increased expression of PDL2 but not of PDL1 (Fig 3A). Stimulation of BMDC from DKO mice with LPS further increased the levels of PDL2, Class II MHC, and CD86 (Fig 3B).

In view of the increased ability of DKO DCs to upregulate IL-10 *in vivo* we also evaluated IL-10 expression in wt and DKO BMDCs. Stimulation of DKO BMDC with LPS resulted in higher IL-10 expression as compared to LPS-treated wt BMDCs (Fig 3C). Increased upregulation of IL-10 in DKO BMDCs was detected by QPCR but not ELISA, possibly due to differences in the kinetics of IL-10 production in DKO DCs. We next investigated whether the hyper-responsiveness of DKO BMDCs to TLR4 stimulation would extend to IFN**β**, another key cytokine produced by DCs, which together with IL-10, has been implicated in lupus pathogenesis (Fig 3C). LPS stimulation led to higher levels of IFN**β** expression in DKO BMDCs than in wt BMDCs, which could be observed by both QPCR and ELISA. Increased IL-10 and IFN**β** expression was primarily observed in BMDCs lacking both DEF6 and SWAP-70 but not in BMDCs lacking either DEF6 or SWAP-70 alone (S3 Fig). Thus DKO BMDCs exhibit increased hyper-responsiveness to TLR4 stimulation.

Given the importance of endosomal TLRs such as TLR9 and TLR7 in DC dysfunction in SLE [44, 45], DKO BMDCs were also stimulated with TLR9 and TLR7 ligands. Exposure of DKO BMDCs to CpG again led to higher expression of both IL-10 and IFN**β** as compared to wt BMDCs as assessed by both QPCR and ELISA (Fig 3D). Similar results were also obtained with imiquimod although TLR7 stimulation was not as effective as CpG in promoting IL-10 and IFN**β** production by BMDCs (S3 Fig). We also took advantage of the reporter mice to assess whether the increased IL-10 production by DKO BMDCs was due to the higher frequency of mature DCs in DKO cultures (Fig 3E). Increased levels of Thy1.1 expression were observed in wt and DKO BMDCs upon CpG and, to a lesser extent, upon LPS stimulation within both the MHCII Hi and the MHCII lo populations suggesting that their enhanced ability to respond to TLR signals is not simply due to an increase in spontaneous maturation. Thus, DKO BMDCs exhibit a broad hyper-responsiveness to TLR ligands as evidenced by enhanced expression of both IL-10 and IFN**β**.

### Selective requirements for IRF4 in DKO DC dysfunction

The dysregulated IL-10 production exhibited by the DKO DCs in response to TLRs prompted us to assess the expression of IRF4 in these DCs. Wt and DKO BMDCs exhibited similar levels of IRF4 expression in the absence of stimulation (S4 Fig). Exposure to LPS, CpG, or imiquimod upregulated IRF4 expression to similar levels in wt and DKO BMDCs (S4 Fig). Expression of IRF8 was also equivalent between wt and DKO BMDCs and was largely unaffected by TLR stimulation (S4 Fig). Thus, consistent with the idea that DEF6 and SWAP-70 primarily regulate the activity of IRF4, DKO BMDCs do not exhibit abnormalities in IRF4 expression.

**Fig 4:**
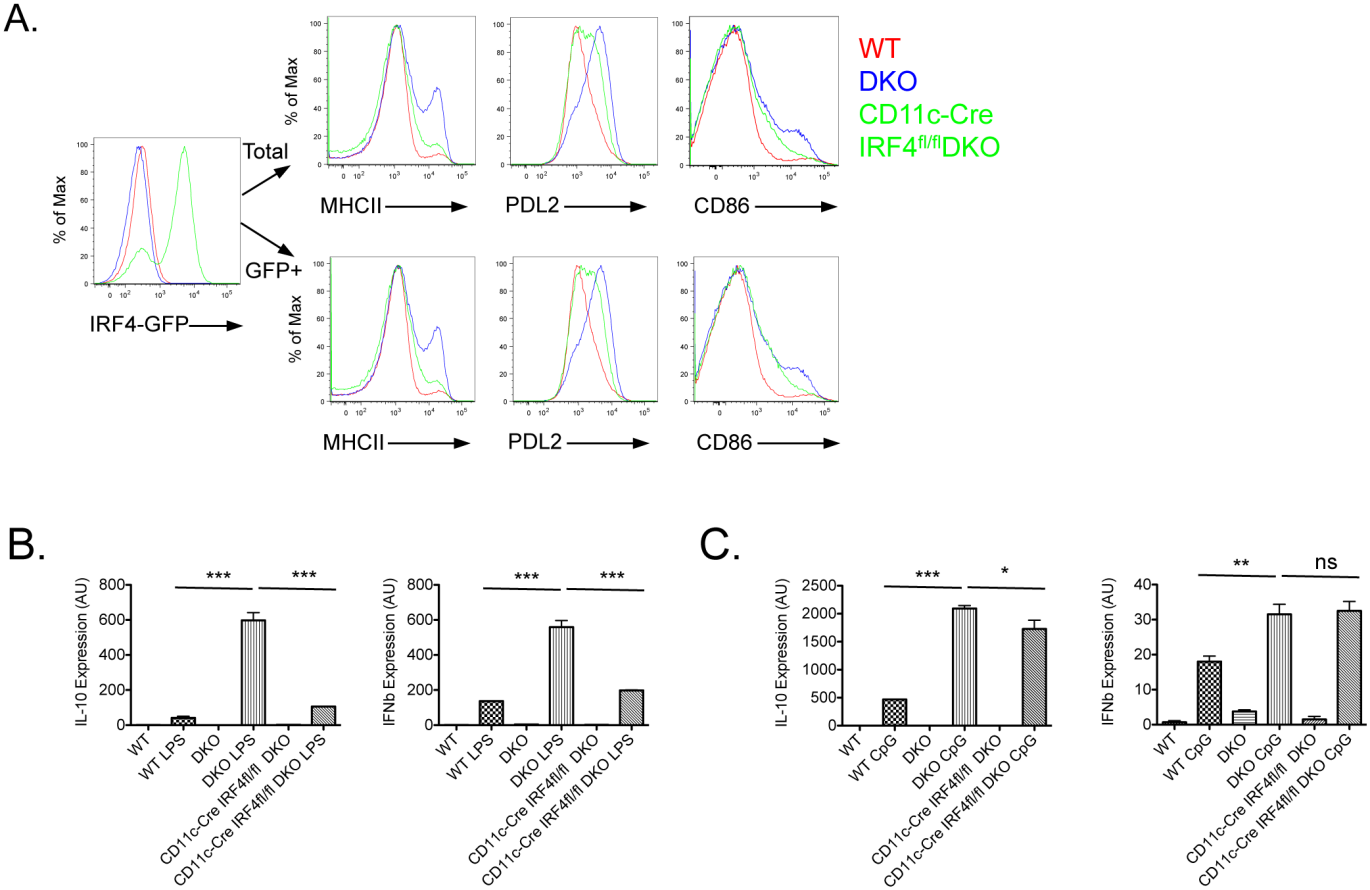
Selective requirement for IRF4 in TLR stimulated DKO BMDCs. (**A**) WT, DKO and CD11c-Cre IRF4^fl/fl^ DKO BMDCs were generated *in vitro* in presence of GM-CSF for 7–9 days prior to FACS analysis of MHCII, CD86 and PDL2. Histograms show relative expression of the indicated marker on WT (red), DKO (blue) and CD11c-Cre IRF4^fl/fl^ DKO (green) total CD11c^+^DCs or GFP^+^CD11c^+^DCs. One representative experiment out of 3 independent experiments is shown. **(B-C)** BMDCs were generated *in vitro* for 7 days. CD11c^+^DCs were purified by magnetic sorting followed by in vitro stimulation with 0.1**μ**g/ml LPS or 3**μ**M CpG, for 24h. IL-10 and IFN**β** gene expression in LPS treated (**□**) or CpG treated (**C**) WT, DKO and CD11c-Cre IRF4^fl/fl^ DKO BMDCs were assayed by qPCR. One representative experiment out of 4 independent experiments is shown. *: p<0.05; **: p<0.01, ***: p<0.001.

To directly assay the contribution of IRF4 to the DC abnormalities observed in DKO mice we selectively deleted this transcription factor in DKO DCs by crossing DKO mice with CD11c-Cre IRF4^fl/fl^ mice (S4 Fig). Generation of BMDC from CD11c-Cre IRF4^fl/fl^ DKO mice appeared to be normal as assessed by CD11c expression (S4 Fig). The lack of IRF4 decreased but not completely corrected the higher levels of Class II MHC, PDL2 and CD86 observed in DKO DCs (Fig 4A). The inability to observe a more marked downregulation in PDL2 and CD86 on DCs from CD11c-Cre IRF4^fl/fl^ DKO mice was not due to an inability to fully delete IRF4. Indeed similar results were obtained by gating on IRF4 deleted BMDCs, which could be evaluated by taking advantage of the fact that deletion of IRF4 is accompanied by the induction of GFP [8]. These findings suggest that the upregulation of CD86, PDL2, and Class II MHC in DKO BMDCs is partly but not exclusively regulated by IRF4.

To evaluate the effects of the lack of IRF4 on the production of IL-10 and IFN**β** by DKO BMDCs, we stimulated BMDCs derived from wt, DKO, or CD11c-Cre IRF4^fl/fl^ DKO mice with LPS for 24 hrs (Fig 4B). IL-10 and IFN**β** expression was then assayed by QPCR. Presence of IRF4 was necessary for the increased expression of both IL-10 and IFN**β** mRNAs upon stimulation with LPS. In contrast to the LPS cultures, the enhanced production of IL-10 and IFN**β** exhibited by DKO BMDCs upon stimulation with CpG was completely unaffected by the lack of IRF4 (Fig 4C). Thus the hyper-responsiveness of DKO BMDCs to TLR4 and TLR9 ligands is mediated by IRF4-dependent and IRF4-independent pathways, respectively.

**Fig 5:**
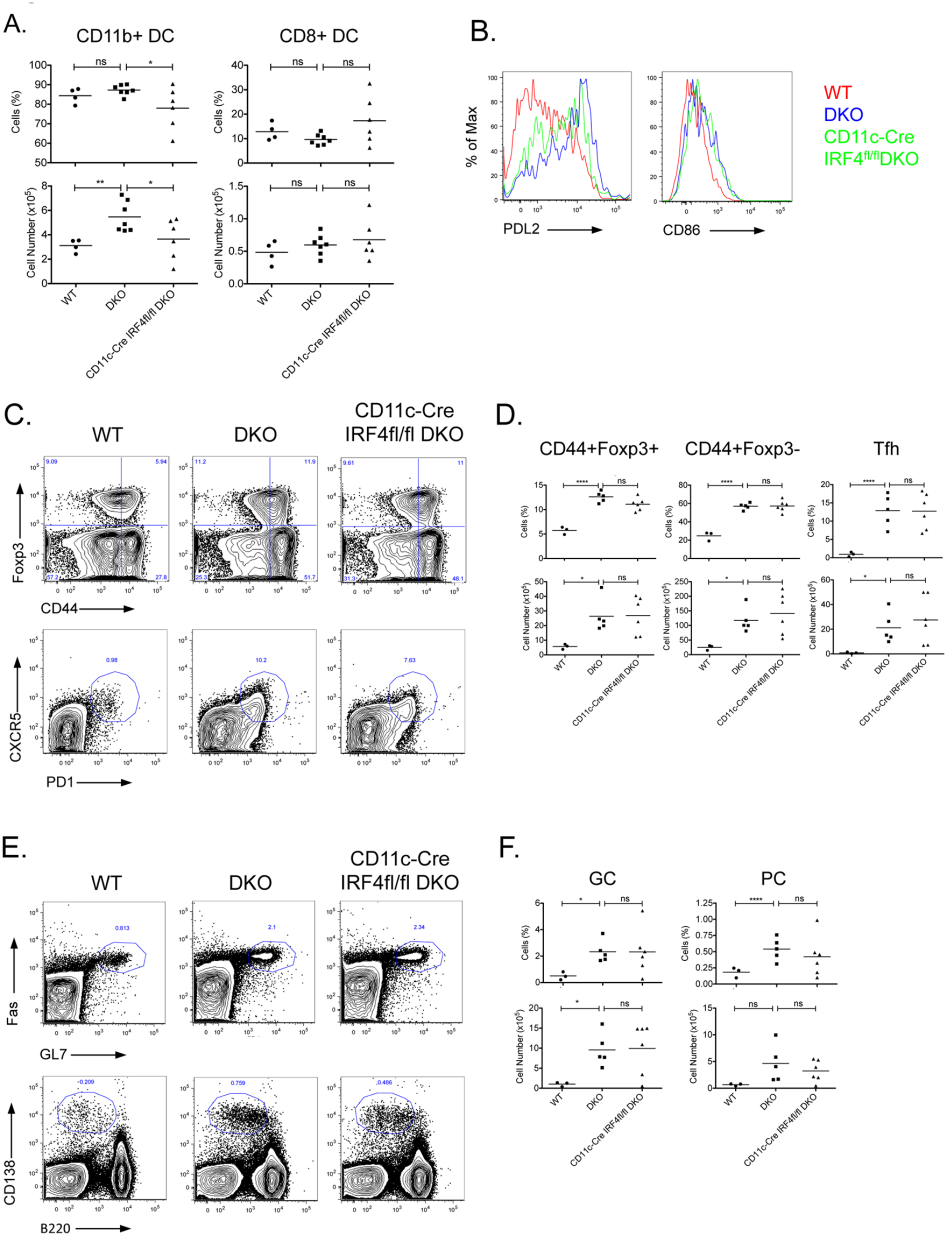
Effects of IRF4 deletion in CD11c^+^ cells on DC, T, and B cell populations in DKO mice. (**A)** Spleens of 14–20 weeks old WT, DKO and CD11c-Cre IRF4^fl/fl^ DKO mice were assayed for DC populations by flow cytometry. Splenocytes were gated on MHCII^+^CD11c^+^B220^-^ conventional DCs and analyzed for the proportion and numbers of CD11b^+^DCs and CD8^+^DCs. (**B**) CD86 and PDL2 cell surface expression on CD11b^+^DCs. Histograms show relative expression of the indicated marker on WT (red), DKO (blue) and CD11c-Cre IRF4^fl/fl^ DKO (green) mice. Representative data of at least 2 independent experiments with a total of 3–5 mice per group is shown. (**C-F**) WT, DKO and CD11c-Cre IRF4^fl/fl^ DKO total splenocytes were analyzed for their CD4^+^T cell (**C-D**) and B cell (**E-F**) populations. Percentages and numbers of activated Tregs (CD4^+^Foxp3^+^CD44^+^), activated T cells (CD4^+^Foxp3^-^CD44^+^), Tfh (CD4^+^Foxp3^-^PD1^+^CXCR5^+^), germinal center B cells (GC; B220^+^GL7^+^Fas^+^) and plasma cells (PC; B220^+^CD138^+^) are shown. Scatter plots show data of individual mice and mean value of 4 independent experiments. *: p<0.05; **: p<0.01, ***: p<0.001, ****: p≤0.0001.

We next conducted an analysis of the effects of IRF4 deletion on the DC populations in DKO mice (Fig 5A and S5 Fig). Deletion of IRF4 using the CD11c-Cre transgene led to a decrease in the CD11b^+^ DC population without exerting any significant effects on the CD8^+^ DC subset. Surprisingly, the lack of IRF4 did not significantly affect the upregulation of PDL2 and CD86 on DKO DCs (Fig 5B) suggesting that upregulation of these markers on CD11b^+^ DCs can occur in the absence of IRF4. An analysis of the T cell compartment in DKO mice demonstrated that the absence of IRF4 in CD11c^+^ cells did not significantly alter the abnormal expansion of T_FH_ cells or Tregs that occurs in these mice (Fig 5C and 5D). The increased frequencies of GC B cells and plasma cells in these mice were also not diminished by the deletion of IRF4 (Fig 5E and 5F). Thus, the presence of IRF4 in DCs does not significantly impact the aberrant T and B responses observed in the DKO mice.

## Discussion

Although the IRF family of transcription factors plays a central role in the physiological control of DC differentiation and function [4, 19], their precise contribution to DC dysfunction in complex inflammatory settings like those encountered in autoimmune disorders has not been delineated. In particular, IRF4 has been shown to be critical for the function of DC subsets that can promote either T_H_-17 or T_H_2 responses [23–26]. Here we have employed mice that lack the expression of DEF6 and SWAP-70, two molecules known to restrain IRF4 function in T and B cells [28], to explore the contribution of DC-IRF4 to the lupus-like syndrome that develops in these mice. We demonstrate that CD11b^+^ DCs from DKO mice exhibit enhanced expression of PDL2 and dysregulated IL-10 production *in vivo* and *in vitro*. In line with the known involvement of IRFs in mediating TLR signals, we also show that DKO DCs are hyper-responsive to several TLR ligands. Notably, ablation of IRF4 in DKO DCs was effective in ameliorating TLR4- but not TLR9-mediated hyper-responsiveness indicating that DC dysfunction in this lupus model may encompass both IRF4-dependent and IRF4-independent pathways.

The employment of a dual reporter mouse where Blimp1 and IL-10 competency could be simultaneously evaluated [39, 40] allowed us to obtain unique insights into the differential ability of distinct DC subsets to express these crucial molecules in the context of a lupus model. In line with previous studies in MRL/lpr mice, IL-10 expression could be detected in both DCs and macrophages [43]. Interestingly, however, splenic CD11b^+^ DKO DCs expressed both IL-10 and Blimp1 while increased expression of IL-10 by CD11b^+^ DKO DCs from SDLNs was not accompanied by induction of Blimp1. Given that IRF4 and Blimp1 can cooperate in promoting IL-10 production [17], these findings suggest that regional factors can modulate the precise transcriptional requirements employed by DCs to regulate IL-10 expression. Thus the transcriptional machinery employed by DCs to regulate IL-10 production is endowed with a high degree of plasticity potentially enabling DCs to fine-tune their response to the local inflammatory milieu they face.

The notion that regional differences in the inflammatory environment can regulate the molecular machinery of DCs has recently been shown to be a critical aspect of the regulation of Blimp1 expression in this cell type since expression of the Blimp1-YFP reporter in non-autoimmune mice is observed in subsets of intestinal DCs but not in splenic DCs [46]. In line with those results, we did not detect strong upregulation of Blimp1 expression in splenic DCs from wt mice but only from DKO mice. The finding that splenic DKO DCs expressed high-levels of Blimp1 was furthermore surprising in view of previous studies linking Blimp1 expression in DCs to a tolerogenic phenotype and showing that female mice lacking Blimp1 in DCs develop lupus-like features [47]. It is thus possible that DKO DCs do not contribute to disease initiation and that their upregulation of Blimp1 and IL-10 may represent a compensatory mechanism geared to dampening the autoimmune diathesis. Consistent with this notion, the DC phenotype in DKO mice was not observed at 8 wks of age when the earliest cellular abnormalities (e.g. expansion of T_FH_ cells) can already be detected but correlated with disease progression. Furthermore, while CD11c-Cre IRF4^fl/fl^ DKO mice display a reduction in CD11b^+^ DCs, these mice did not exhibit any decreases in T_FH_ accumulation, germinal center B cell expansion, or plasma cell development and a preliminary analysis in 14–20 wks old mice failed to detect differences in anti-dsDNA antibodies. Alternatively, as reported for MRL/lpr lupus mice [48, 49], the altered DC phenotype observed in DKO mice could be involved in promoting end-organ damage. Although DKO mice exhibit immune-complex deposition in the kidneys they develop only moderate glomerulonephritis by 24 wks of age and do not exhibit accelerated mortality [50]. The generation of additional DKO models where disease is accelerated and/or more severe will thus facilitate a precise dissection of the contribution of DCs to the development of target organ damage in these mice.

Similar to other lupus-prone mice [51], DCs from DKO mice were hyper-responsive to TLR stimulation. This hyper-responsiveness encompassed dysregulated production of both IL-10 and IFN**β**, two cytokines known to play a pathogenic role in SLE [52, 53]. Although this dysregulation could be observed upon stimulation with different TLR ligands, deletion of IRF4 in DKO DCs revealed that this phenotype is driven by an unexpected degree of molecular heterogeneity whereby the concomitant lack of IRF4 corrected the TLR4-mediated hyper-responsiveness but exerted no effects on the TLR9-induced dysfunction. This heterogeneity may enable DCs to tightly modulate the levels and timing of cytokine production in response to different TLR ligands. Indeed, IL-10 production by DKO BMDCs upon exposure to LPS was less robust than that observed upon stimulation with CpG. Since a recent study has revealed that GM-CSF derived BMDCs contain two cell populations with distinct properties [54], we cannot rule out that the differential requirement for IRF4 upon exposure of BMDCs to TLR4 or TLR9 ligands also reflects a differential contribution of these two populations.

At a molecular level, the employment of IRF4-dependent and IRF4-independent pathways by different TLRs is likely linked to their ability to engage distinct IRFs, which, due to their high degree of homology, can then drive the expression of similar targets. Indeed IRF5 has recently been implicated in the regulation of IL-10 production by DCs in response to TLR9 [55]. The ability of distinct IRFs to compensate for each other may also be responsible for the finding that PDL2 expression on DKO DCs was only partially decreased by the lack of IRF4 *in vitro*. Even more striking was the finding that PDL2 expression on DKO DCs *in vivo* was largely unaffected by the lack of IRF4 suggesting that compensation between different IRFs may be even more profound in the presence of the complex inflammatory environment observed in lupus.

## Supporting Information

S1 Fig Relative expansion of CD11b^+^ DCs but not pDCs in DKO mice. (**A**) WT and DKO BMDCs were generated *in vitro* in presence of GM-CSF for 7 days followed by LPS 0.1**μ**g/ml, CpG 3**μ**M or Imiquimod 3**μ**g/ml stimulation for 24 hours. Whole cell extracts were prepared and DEF6, SWAP-70 and **β**-Actin expression analyzed by western blot. A representative blot of two independent experiments is shown. **B**) Spleens of 8 weeks old WT and DKO mice were assayed for DC populations by flow cytometry. Splenocytes were gated on MHCII^+^CD11c^+^ conventional DCs and analyzed for numbers of CD8^+^DCs and CD11b^+^DCs. (**C**) Percentage and number of CD11c^+^PDCA-1^+^ splenic plasmacytoid dendritic cells were analyzed after gating on the B220^+^ population. (**D**) Skin draining lymph nodes from 8 weeks old mice were assayed for DC population numbers by flow cytometry. Cells were gated on MHCII^+^CD11c^+^ conventional DCs and analyzed for numbers of CD8^+^DCs and CD11b^+^DCs. MHCII^Hi^CD11c^+^ migratory DCs were also examined. **(E)** Percentages and numbers of CD11c^+^PDCA-1^+^ splenic plasmacytoid dendritic cells were analyzed in >24 weeks old mice after gating on the B220^+^ population. CD86 and PDL2 cell surface expression in WT (red) and DKO (blue) mice was analyzed. Histograms show relative expression of the indicated marker. Representative data of 2 independent experiments is shown. Scatter plots show data of individual mice and mean value of at least 3 independent experiments. *: p<0.05.
(TIF)

S2 Fig IL-10 production by macrophages in DKO mice. Spleen (**A**) and skin draining lymph nodes (**B**) from 14–16 weeks old WT and DKO IL-10 and Blimp1 dual reporter mice were examined by flow cytometry. Splenocytes were gated on CD11b^+^CD11c^-^B220^-^ macrophages followed by analysis of YFP (Blimp1) and Thy1.1 (IL-10) expression. Percentages and numbers of Thy1.1^+^YFP^-^, Thy1.1^+^YFP^+^ and Thy1.1^-^YFP^+^ cells are shown. Scatter plots show data of individual mice and mean value of 4 independent experiments. *: p<0.05; ***: p<0.001.
(TIF)

S3 Fig IL-10 and IFNβ expression by BMDCs from Swap70^-/-^ and Def6^-/-^ mice. WT, Swap70^-/-^, Def6^-/-^ and DKO BMDCs were generated *in vitro* for 7 days. CD11c^+^DCs were purified by magnetic sorting followed by in vitro stimulation with 0.1**μ**g/ml LPS or 3**μ**g/ml of Imiquimod for 24h. (**A**) IL-10 and IFN**β** gene expression in LPS treated WT, Swap70^-/-^, Def6^-/-^ and DKO BMDCs were assayed by qPCR. One representative experiment out of 2 independent experiments is shown. (**B**) Purified CD11c^+^ DC were stimulated with Imiquimod for 24 hours and IL-10 and IFN**β** gene expression evaluated by qPCR. One representative experiment out of at least 3 independent experiments is shown. *: p<0.05; ***: p<0.001.
(TIF)

S4 Fig Expression of different IRF family members in DKO BMDCs. (**A**) WT and DKO CD11c^+^BMDCs were stimulated *in vitro* with 0.1**μ**g/ml LPS, 3**μ**M CpG or 3**μ**g/ml Imiquimod for 24h. Whole cell extracts were prepared and analyzed for IRF4, IRF8 and **β**-actin expression by western blot. Representative blot of at least 2 independent experiments is shown. (**B**) WT, DKO and CD11c-Cre IRF4^fl/fl^ DKO BMDCs were generated *in vitro* in presence of GM-CSF for 7 days. CD11c^+^ DCs were further purified by magnetic sorting followed by in vitro culture without stimulation for 24h. Cells were harvested and RNA prepared for IRF4 gene expression analysis by qPCR. Data represent normalized expression values relative to WT mice. One representative experiment out of 4 independent experiments is shown. ***: p<0.001. (**C**) Alternatively cells were analyzed by FACS for CD11c expression. Histogram shows relative expression of CD11c on DKO (blue) and CD11c-Cre IRF4^fl/fl^ DKO (green) BMDCs. One representative experiment out of 3 independent experiments is shown.
(TIF)

S5 Fig IRF4 deletion in DCs from CD11c-Cre IRF4^fl/fl^ DKO mice. (**A)** Spleens from 14–20 weeks old WT, DKO and CD11c-Cre IRF4^fl/fl^ DKO mice were assayed for IRF4 deletion in DC populations by flow cytometry. Splenocytes were gated on MHCII^+^CD11c^+^B220^-^ conventional DCs and analyzed for GFP expression in CD8^+^DCs and CD11b^+^DCs. Histograms show relative GFP expression on WT (red), DKO (blue) and CD11c-Cre IRF4^fl/fl^ DKO (green) mice. Representative data of 4 independent experiments is shown.
(TIF)
