# Supplementary figures and images for: IRF4-Dependent and IRF4-Independent Pathways Contribute to DC Dysfunction in Lupus
Source: PLoS One. 2015 Nov 6;10(11):e0141927. doi: 10.1371/journal.pone.0141927 (PMC4636285; doi:10.1371/journal.pone.0141927)

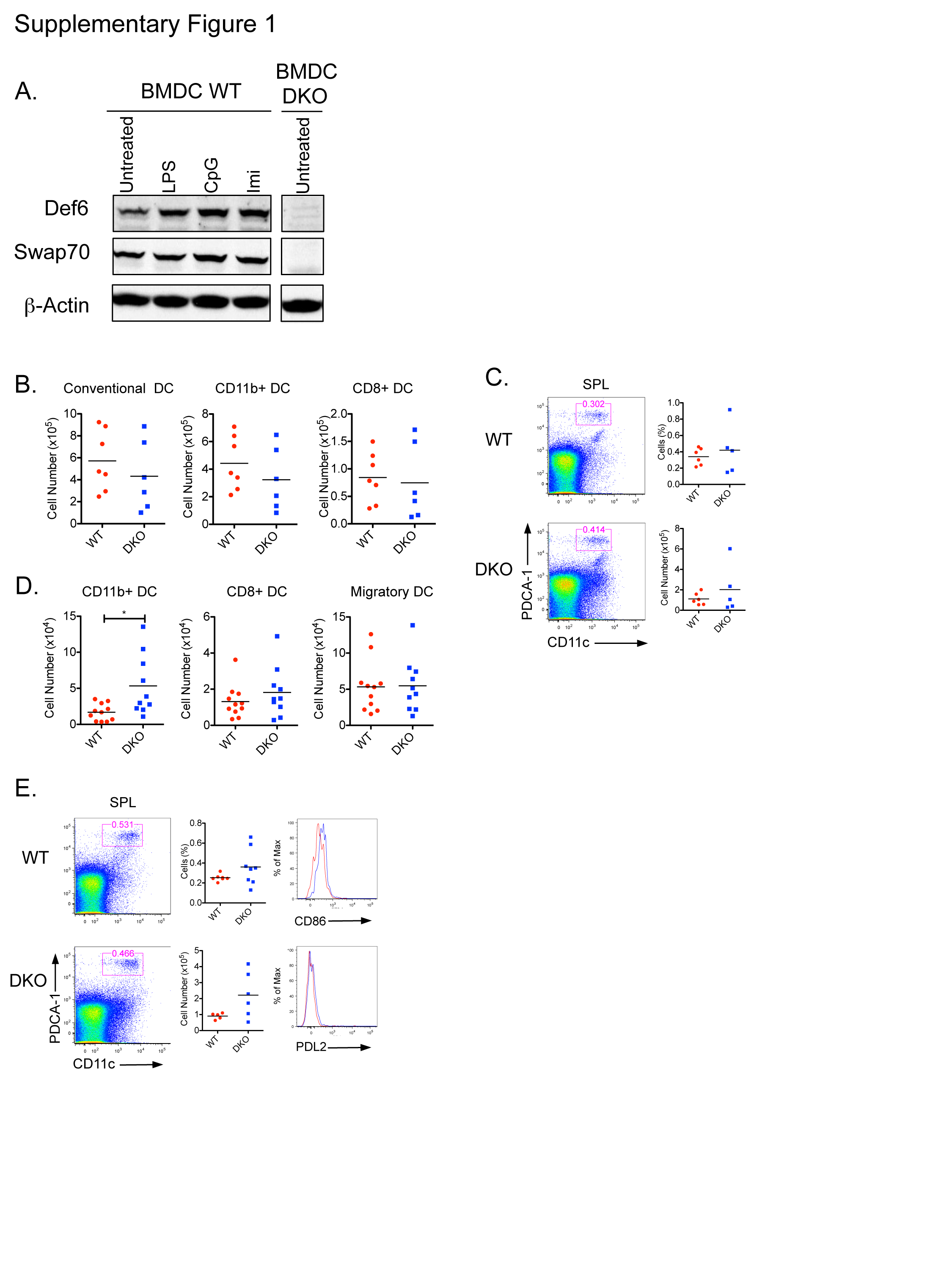

Supplement: S1 Fig — (A) WT and DKO BMDCs were generated in vitro in presence of GM-CSF for 7 days followed by LPS 0.1μg/ml, CpG 3μM or Imiquimod 3μg/ml stimulation for 24 hours. Whole cell extracts were prepared and DEF6, SWAP-70 and β-Actin expression analyzed by western blot. A representative blot of two independent experiments is shown. B) Spleens of 8 weeks old WT and DKO mice were assayed for DC populations by flow cytometry. Splenocytes were gated on MHCII+CD11c+ conventional DCs and analyzed for numbers of CD8+DCs and CD11b+DCs. (C) Percentage and number of CD11c+PDCA-1+ splenic plasmacytoid dendritic cells were analyzed after gating on the B220+ population. (D) Skin draining lymph nodes from 8 weeks old mice were assayed for DC population numbers by flow cytometry. Cells were gated on MHCII+CD11c+ conventional DCs and analyzed for numbers of CD8+DCs and CD11b+DCs. MHCIIHiCD11c+ migratory DCs were also examined. (E) Percentages and numbers of CD11c+PDCA-1+ splenic plasmacytoid dendritic cells were analyzed in >24 weeks old mice after gating on the B220+ population. CD86 and PDL2 cell surface expression in WT (red) and DKO (blue) mice was analyzed. Histograms show relative expression of the indicated marker. Representative data of 2 independent experiments is shown. Scatter plots show data of individual mice and mean value of at least 3 independent experiments. *: p<0.05. (TIF) [file pone.0141927.s001.tif]

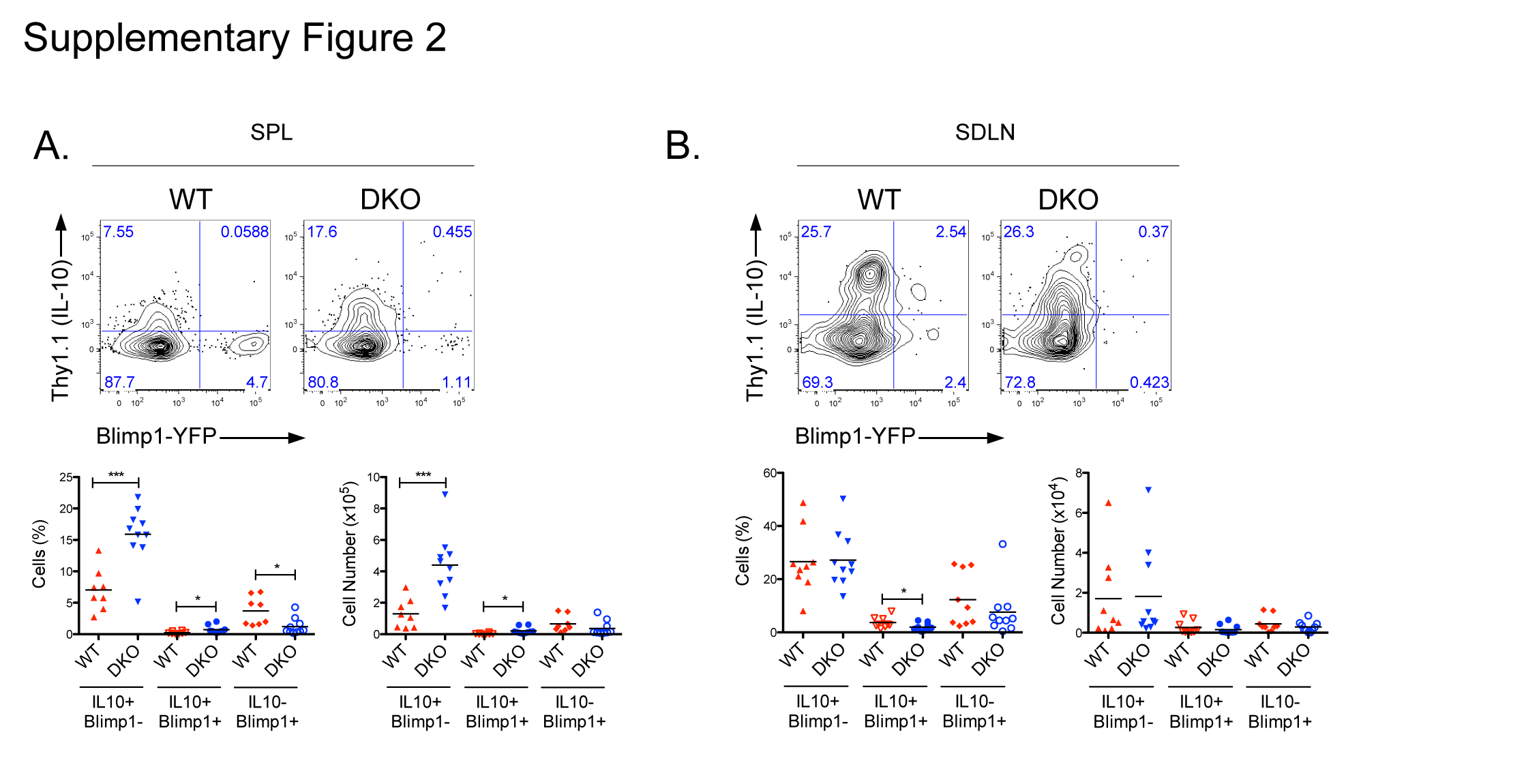

Supplement: S2 Fig — Spleen (A) and skin draining lymph nodes (B) from 14–16 weeks old WT and DKO IL-10 and Blimp1 dual reporter mice were examined by flow cytometry. Splenocytes were gated on CD11b+CD11c-B220- macrophages followed by analysis of YFP (Blimp1) and Thy1.1 (IL-10) expression. Percentages and numbers of Thy1.1+YFP-, Thy1.1+YFP+ and Thy1.1-YFP+ cells are shown. Scatter plots show data of individual mice and mean value of 4 independent experiments. *: p<0.05; ***: p<0.001. (TIF) [file pone.0141927.s002.tif]

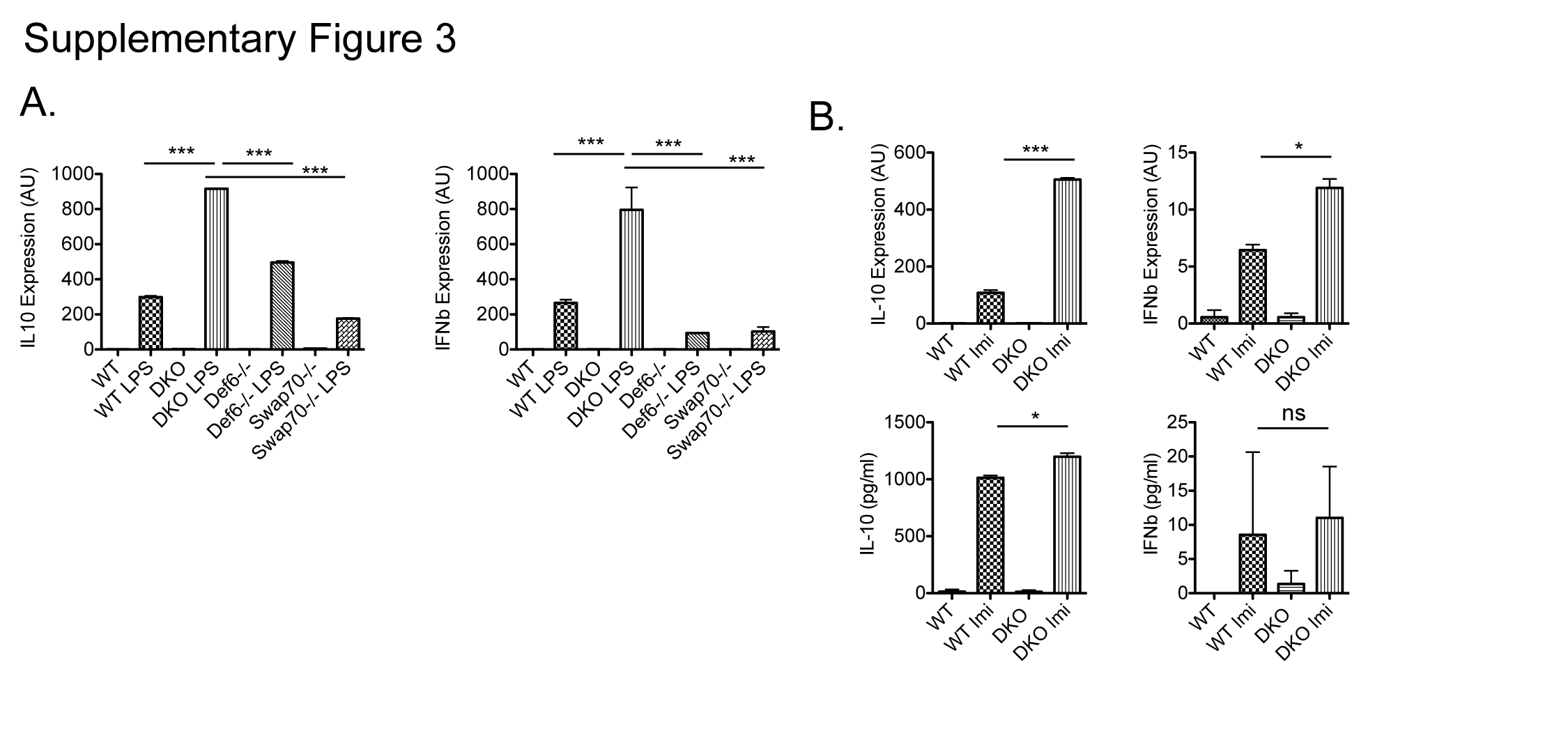

Supplement: S3 Fig — WT, Swap70-/-, Def6-/- and DKO BMDCs were generated in vitro for 7 days. CD11c+DCs were purified by magnetic sorting followed by in vitro stimulation with 0.1μg/ml LPS or 3μg/ml of Imiquimod for 24h. (A) IL-10 and IFNβ gene expression in LPS treated WT, Swap70-/-, Def6-/- and DKO BMDCs were assayed by qPCR. One representative experiment out of 2 independent experiments is shown. (B) Purified CD11c+ DC were stimulated with Imiquimod for 24 hours and IL-10 and IFNβ gene expression evaluated by qPCR. One representative experiment out of at least 3 independent experiments is shown. *: p<0.05; ***: p<0.001. (TIF) [file pone.0141927.s003.tif]

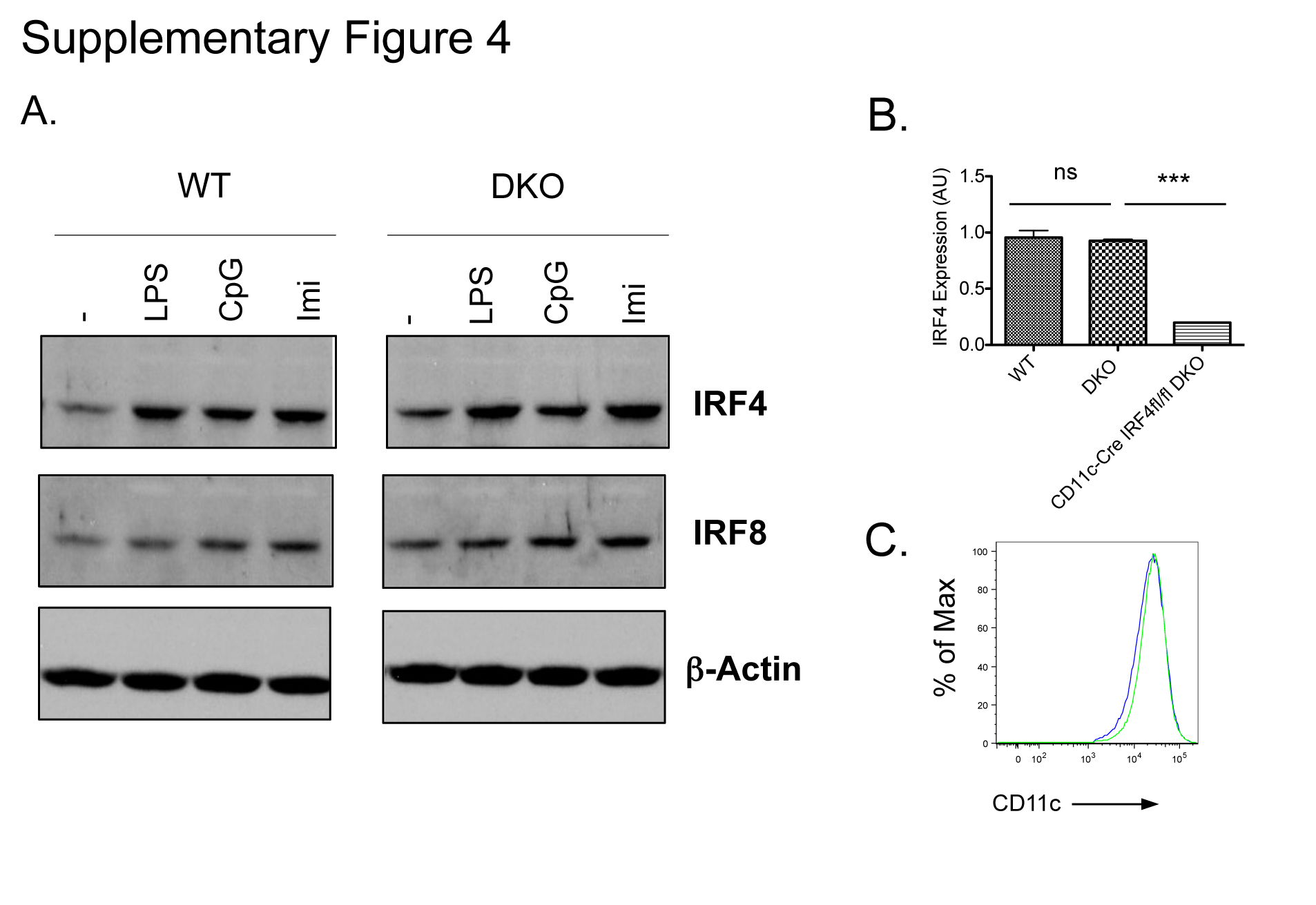

Supplement: S4 Fig — (A) WT and DKO CD11c+BMDCs were stimulated in vitro with 0.1μg/ml LPS, 3μM CpG or 3μg/ml Imiquimod for 24h. Whole cell extracts were prepared and analyzed for IRF4, IRF8 and β-actin expression by western blot. Representative blot of at least 2 independent experiments is shown. (B) WT, DKO and CD11c-Cre IRF4fl/fl DKO BMDCs were generated in vitro in presence of GM-CSF for 7 days. CD11c+ DCs were further purified by magnetic sorting followed by in vitro culture without stimulation for 24h. Cells were harvested and RNA prepared for IRF4 gene expression analysis by qPCR. Data represent normalized expression values relative to WT mice. One representative experiment out of 4 independent experiments is shown. ***: p<0.001. (C) Alternatively cells were analyzed by FACS for CD11c expression. Histogram shows relative expression of CD11c on DKO (blue) and CD11c-Cre IRF4fl/fl DKO (green) BMDCs. One representative experiment out of 3 independent experiments is shown. (TIF) [file pone.0141927.s004.tif]

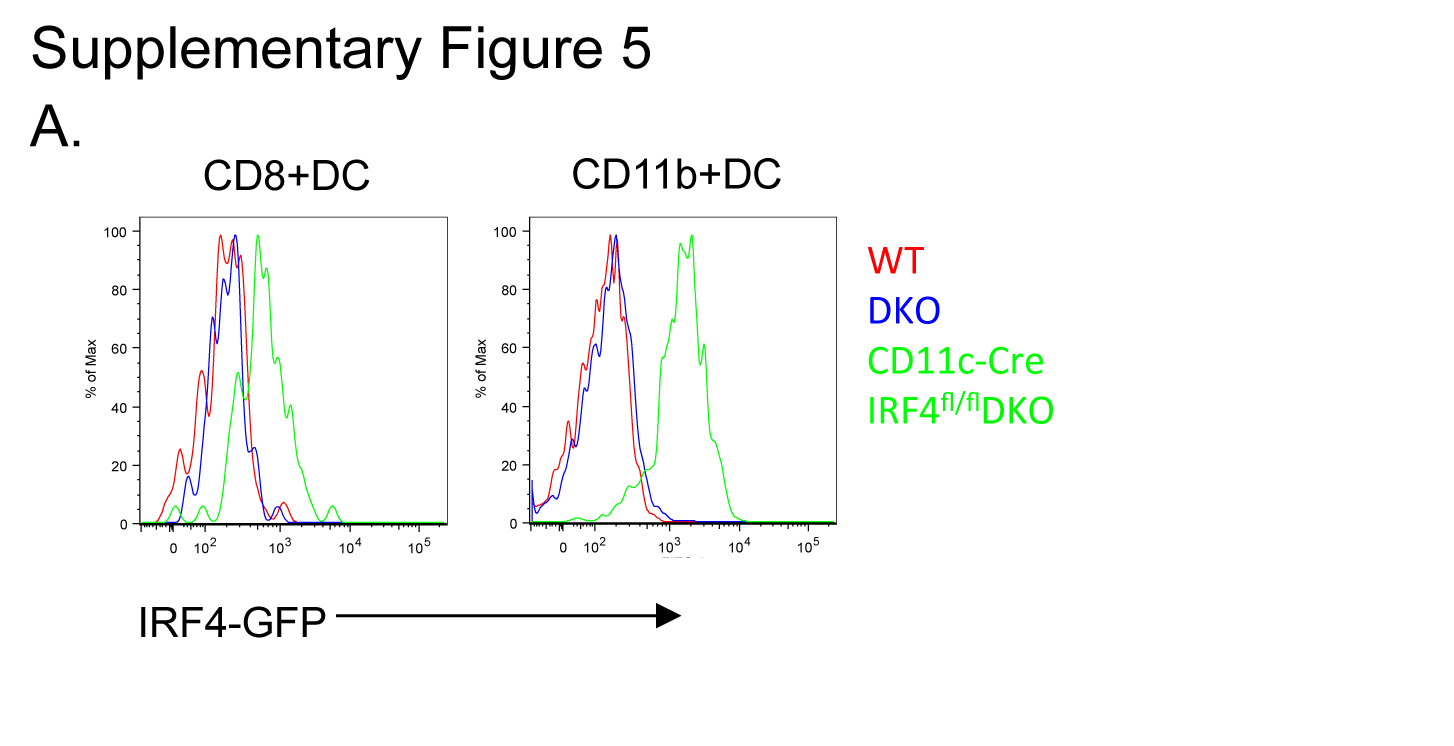

Supplement: S5 Fig — (A) Spleens from 14–20 weeks old WT, DKO and CD11c-Cre IRF4fl/fl DKO mice were assayed for IRF4 deletion in DC populations by flow cytometry. Splenocytes were gated on MHCII+CD11c+B220- conventional DCs and analyzed for GFP expression in CD8+DCs and CD11b+DCs. Histograms show relative GFP expression on WT (red), DKO (blue) and CD11c-Cre IRF4fl/fl DKO (green) mice. Representative data of 4 independent experiments is shown. (TIF) [file pone.0141927.s005.tif]
